# Importance of Protected Areas for Biodiversity Conservation in Central Côte D'ivoire: Comparison of Termite Assemblages between Two Neighboring Areas Under Differing Levels of Disturbance

**DOI:** 10.1673/031.012.13101

**Published:** 2012-11-10

**Authors:** Kanvaly Dosso, Kolo Yéo, Souleymane Konaté, Karl Eduard Linsenmair

**Affiliations:** ^1^ Station d'Ecologie de Lamto, Université Nangui Abrogoua, Côte d'Ivoire; ^2^ Department of Animal Ecology and Tropical Biology, University of Würzburg, Germany; ^3^ BP 28 N'Douci, Côte d'Ivoire

**Keywords:** burned savanna, forest, Lamto Reserve, relative abundance, rural domain, savanna woodland, species composition, species richness

## Abstract

To highlight human impact on biodiversity in the Lamto region, termites were studied with regard to their use as bio-indicators of habitat change in the tropics. Using a standardized method, termites were sampled in the three most common habitat types, i.e., in semi-deciduous forest, savanna woodland, and annually burned savanna, all inside Lamto Reserve and its surrounding rural domain. Termite species richness fell from 25 species in the Lamto forest to 13 species in the rural area, involving strong modification in the species composition (species turnover = 59 %). In contrast, no significant change in diversity was found between the Lamto savannas and the rural ones. In addition, the relative abundance of termites showed a significantly greater decline in the rural domain, even in the species *Ancistrotermes cavithorax* (Sjöstedt) (Isoptera: Termitidae), which is known to be ecologically especially versatile. Overall, the findings of this study suggest further investigation around Lamto Reserve on the impact of human activities on biodiversity, focusing on forest conversion to land uses (e.g. agricultural and silvicultural systems).

## Introduction

Changes in landscape structure alter the population dynamics and composition of the concerned communities (Vasconcelos 1999; Barros et al. 2002; Mathieu et al. 2005). Habitat loss is considered a major threat to global biodiversity (Fahrig 2002; Brooks et al. 2002), increasing the extinction rate of species in most ecosystems (Brooks et al. 2002). This loss is particularly intense in the tropics, where many tropical forests are replaced with agricultural and silvicultural systems (Tilman et al. 2001). Habitat conversion leads to a simplification of natural communities, resulting in the promotion of species that are tolerant to altered environments. Furthermore, it is responsible for the elimination of many specialist species (Heywood and Watson 1995; Boren et al. 1999; Hansen and Rotella 2002). In the Lamto region, such damages are essentially due to human activities such as lighting of bush fires, land-conversion and land-use, hunting, harvesting of firewood, and logging. While man-made fires contribute essentially to landscape structuring inside Lamto Reserve, the combination of the mentioned activities is considered responsible for habitat change and habitat destruction, respectively, in the adjacent rural domain. Despite the negative consequences these anthropogenic impacts may exert on the biodiversity of tropical soil organisms, only a few studies have focused on arthropods in general and on termites in particular. Termites belong to the most dominant macroarthropod detritivores in the tropics and they are particularly diverse and abundant (Wood and Sands 1978; Collins 1983; Eggleton et al. 1999; Eggleton 2000). They are major agents in decomposition processes, and play an important part in nutrient and carbon fluxes and cycles (Lawton et al. 1996; Bignell et al. 1997; Tayasu et al. 1997). Their activities, such as soil feeding, subterranean tunneling, and mound building, maintain the very important macropore structure, redistribute organic matter, improve soil stability and quality, and improve water absorbing and storing capacity (Lee and Wood 1971; Holt and Lepage 2000). Given the central role of the decomposer food web in regulating plant growth (Laakso and Setälä 1999; Wardle 1999) and the influence that termites have on ecosystem processes (Lavelle et al. 1997; Bignell and Eggleton 2000), it is desirable to understand how habitat disturbance affects their assemblages. While a few studies have demonstrated that termites are sensitive to habitat disturbance (Bignell and Eggleton 2000; Eggleton et al. 2002), only a very limited number of such studies have compared the communities living in areas exposed to different levels of disturbance. The Lamto region offered the opportunity to examine termite assemblages in three differently disturbed habitat types (annually burned savanna, savanna woodland, and semideciduous forest), with each habitat located inside Lamto Reserve as well as in the rural domain. The annually burned savanna possesses a tree cover ranging from 7 to 36% (Gautier 1990). It is a fuel-rich habitat, deliberately burned almost every year. Such a habitat type covers the lower part of slopes and the hydromorphic plateaus (Menaut and Abbadie 2006). As for the savanna woodlands studied, they consisted of grassy or shrubby savanna sections that have randomly escaped the fire for more than five years and were subjected to tree invasion (cf. Vuattoux 1970, 1976). With a tree cover of more than 60%, the vegetation was a mixture of grass, shrub, and tree layers (Gautier 1990). This savanna type appears mostly on slopes (Menaut and César 1979). The impact of fire depends on the strength of the seasonal drought and mainly on the amount of fuel produced since the last fire. The semi-deciduous forests are entirely surrounded by savannas. They possess discontinuous canopies and are partly covered with transitional vegetation comprising both forest and savanna tree species. Inside Lamto Reserve, this forest type is impacted by fire only at its periphery because the grass stratum is lacking, replaced by a thin layer of leaf-litter which does not offer enough fuel to allow the fire to invade deeply inside. Inside the rural domain, however, forests are subjected to strong human pressure due to agricultural use, hunting, logging, and bush fires. After our previous study (cf. Dosso et al. 2010) showing how termite diversity and abundance are developing across fire-induced habitat variability at Lamto, the present study aimed at comparing changes in termite assemblages between the protected area and the neighboring rural domain, which some time ago (around 50 years) was part of the formerly described forest.

## Materials and Methods

### Study site

**Figure 1.**
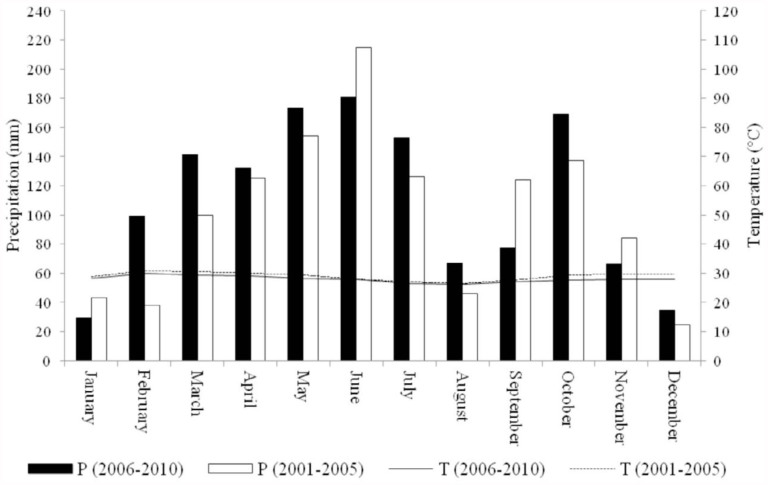
Mean monthly precipitation and temperature in the Lamto region over the study period (2006–2010) and the five years (2001–2005) preceding the samplings (Data source: Lamto Geophysical Station). High quality figures are available online.

Our study was carried out inside Lamto Reserve (6° 13′ N, 5° 02′ W) (in 2006) and in the surrounding rural domain (in 2010). In this part of the country, the vegetation is a typical Guinean savanna, where fire is held responsible for the unexpected presence of savannas where climate should be able to sustain rain forests (Monnier 1968). The ample water availability supports the development of a rich vegetation, which becomes, with drying up, a copious fuel source for the fires occurring every year (usually mid-January). The fires exert a stabilizing effect on the habitat mosaic by preventing massive tree invasion into the savanna, thereby hindering the reestablishment of forests and freezing the forest-savanna boundary in a historical position (Gillon 1983). Although the fires have a strong negative effect on newly recruited woody plants, the regular human-lit fires cannot entirely prevent plant invasions, because they are, as mid-dry-season fires, neither hot nor expansive enough to kill all tree and shrub species before they are tall enough to resist fires (Abbadie et al. 2006). This savanna possesses for most of the year a dense and high grass layer, where scattered trees and bushes vary considerably in their abundance, leading to a high spatial heterogeneity. The annual precipitation reaches 1200 mm, and the dry season usually lasts less than two months (Menaut 1983). Over the period of the study (2006–2010), a long rainy season occurred from February to July, interrupted by a short dry season in August. A short rainy season occurred from September to November, while the driest season occurred from December to January. As for the five years preceding our sampling sessions (2001–2005), the seasonal repartition was globally similar to that described above, except some minor differences: the long rainy season started one month later (March), and the short rainy season seemed wetter. The curves of mean monthly temperature did not show differences between the two periods (2001–2005 and 2006–2010) (Figure 1).

### Sampling design

Termites were sampled using a standardized method designed for rapid assessment of termite diversity by Jones and Eggleton (2000). Five separate blocks of each habitat type were sampled by delimiting at random one transect 100 m long and 2 m wide through each block. As transects were taken as the sampling units, they were oriented so as to cover all heterogeneity within the respective habitats. The blocks of the same habitat type were separated by at least 200 m in such a manner as to be representative of the area. Each transect was subdivided into 20 contiguous quadrats of 10 m^2^ (5 m × 2 m) each in order to standardize the sampling effort. In all quadrats, microhabitats (logs, litter, stumps, twigs, nests, runways sheeting, fallen branches, etc.) were hand-searched up to a height of 2 m above ground level. As the method was designed for use in forests, modifications were made in the savanna by searching for termites between grass tufts or by uprooting grass tufts. All termites encountered were collected. Twelve samples of surface soil (each about 12 cm × 12 cm to 10 cm depth) were dug out in the quadrat at random locations. The soil was hand-sorted in situ, and a representative sample of termites (around 10 individuals of each caste present) was sorted and put into 70% ethyl alcohol. Termites collected were both of the soldier and worker castes; the alates were excluded because they did not necessarily imply the presence of a viable colony. In order to sample all 20 quadrats in one day, four trained collectors were deployed, with two people at a time sampling 10 quadrats for 30 minutes per quadrat. Following Andersen (1991), samplings were based on the occurrence of individuals (presence-absence) rather than their number, with respect to the social habit of termites. The fieldwork took place during dry seasons, as these periods were especially favourable for sampling. During dry seasons, termite foraging activities are more evident in the study area because the termites consume preferentially dry plant matter. The evident foraging activities enabled an easy collection of a great number of species.

### Identification and feeding group classification

All sampled termites were identified at the Lamto station and confirmed at the Royal Museum for Central Africa, Tervuren, Belgium. Specimens were identified to the level of species or, where species-level identification proved impossible, to numbered morphospecies using standard determination keys such as those of Webb (1961), Bouillon and Mathot (1965, 1966, 1971), and the descriptions made by Grasse (1986). After the identification, each species was placed into one of the feeding groups (i.e., fungus-growers, soil-feeders, wood-feeders, and grass-feeders) defined according to termite diet (Josens 1977), mandible morphology (Deligne 1966), and gut content in the worker caste (Sands 1998). All fungus-growers belong to the subfamily Macrotermitinae. They consume grass, dung, wood, and litter via an exo-symbiosis with the fungus *Termitomyces* for the decomposition of plant matter. The soil-feeders feed on soil organic matter and occasionally on very decayed wood. Most wood-feeders consume dead wood, but some species feed on living plants. The grass-feeders are exclusive consumers of grassy plant materials. The species of the investigated areas belong exclusively to the genus *Trinervitermes.*

### Data analysis

The species richness of termites was determined by enumerating the number of species observed over the whole transect. Because presence-absence data was used, the relative abundance was defined as the number of encounters per transect, where the presence of one species in a quadrat represented one encounter (Magurran 2004). Thus, occurrences were preferred because the number of individuals could be misleading when dealing with social animals such as termites that are patchily distributed (Yéo et al. 2011). Following the description of Colwell and Coddington (1994) for incidence data, the second order and non-parametric estimator Chao2 was used as the estimator of the species richness. It takes into account the distribution of species among quadrats, and it needs for its calculation only the number of species found in just one quadrat and the number of species in exactly two. Simpson's index served to measure the diversity of the termite assemblage. This index and its evenness were computed by using the program “Ecological Methodology” (www.Zoology.ubc.ca/Krebs). To better visualize the similarity of habitat types, the Unweighted Pair-Group clustering method using arithmetic Averages (UPGMA) was performed with the software STATISTICA 7.1 (www.statsoft.com). Then, the complementarity between the assemblages allowed the description of the differences between habitats in terms of their species identity (Vane-Wright et al. 1991). We termed as “dominant species” those whose total number of occurrences equaled or exceeded the mean number of occurrences per habitat (= 20). After testing the homogeneity of variances, variations in termite species richness and abundances were examined using the one-way analysis of variance (ANOVA 1) while LSD (Least Significant Difference) tests were applied to detect differences between the habitat types and study areas.

## Results

### Species richness and species diversity of termites

A total of 32 species were sampled in the two areas combined (Table 1). Two subfamilies of lower termites (Rhinotermitiade), Coptotermitinae (1 species) and Rhinotermitinae (1 species) and four subfamilies of higher termites (Termitidae), Macrotermitinae (13 species), Termitinae (7 species), Apicotermitinae (3 species), and Nasutitermitinae (6 species), were represented. These species belonged to 21 genera and 4 feeding groups. Although most of the species were encountered inside the Lamto Reserve (30 species), the total species richness did not differ statistically from that found inside the rural domain (22 species). The number of sampled species varied between the habitat types. At Lamto, the forest (25 species) was significantly richer than the annually burned savanna and the savanna woodland (18 species) (LSD test, *p* = 0.04, n = 5). In contrast, the highly disturbed rural forest (13 species) was species-poorer than the rural savanna woodland (16 species) and harbored an equal number of species as the annually burned rural savanna (13 species). Simpson's diversity index and its evenness were almost similar for the two areas, with a decreasing trend for the rural domain. Of the characteristics of the termite assemblages, only the relative abundance varied significantly between the Reserve and the rural domain (ANOVA 1, F = 280.9, *p* = 7.10^-5^). According to our criterion, six “dominant” species were recorded for the two areas combined (*Adaiphrotermes* sp. (Isoptera: Termitidae), *Aderitotermes* sp., *Ancistrotermes cavithorax* (Sjöstedt), *Microtermes toumodiensis* (Grassé), *Pseudacanthotermes militaris* Hagan, and *Trinervitermes geminatus* (Wasmann).

### Relative abundance of termites

In the Lamto protected area, 473 species occurrences were registered, representing 64% of the total, as compared to 36% in the rural domain, where 261 occurrences were recorded (Table 3). According to our criteria (termite diet, mandible morphology, and gut content analysis), the species identified were classified into four feeding groups (fungus-growers, soil-feeders, wood-feeders, and grass-feeders). The fungus-growers were the most abundant group in each area investigated. They totaled 295 occurrences out of 473 (62%) and 192 occurrences out of 261 (74%) inside Lamto Reserve and the rural domain respectively. Their total abundance did not vary statistically between the two areas. But, when considering habitat types separately, it was significantly lower in the rural savanna woodland (LSD test, *p* = 0.02, n = 5) compared to the same type of habitat located inside the Reserve. Three of the six “dominant” species (*A. cavithorax*, *M. toumodiensis*, and *P. militaris*) belonged to this feeding group. The abundance of *A. cavithorax* was significantly higher in the Lamto savanna woodland than in the equivalent rural habitat (LSD test, *p* = 0.004, n = 5) and in the Lamto forest than in the rural forest (LSD test, *p* = 0.03, n = 5). As for *P. militaris*, its abundance was significantly higher in the annually burned rural savanna (LSD test, *p* = 0.007, n = 5) and in the rural forest (LSD test, *p* = 0.02, n = 5) than their equivalents located inside Lamto Reserve. While comparing the two study areas, only the abundances of *A. cavithorax* (LSD test, *p* = 0.04, n = 3) and *M. toumodiensis* (LSD test, *p* = 0.03, n = 3) significantly decreased in the rural domain (Table 4). Surprisingly, soil-feeders were more abundant in the annually burned savannas than in the forests. They totaled 98 occurrences out of 473 (21%) and 37 occurrences out of 261 (14%) in the Reserve and the rural domain respectively. At habitat level, they were very significantly less abundant in the rural forest (LSD test, *p* = 3.10^-4^, n = 5) than in the Lamto forest. The “dominant” soil-feeding species were *Adaiphrotermes* sp. and *Aderitotermes* sp., totaling 63 occurrences out of 96 (64%) and 32 occurrences out of 37 (87%) in the Lamto Reserve and the rural domain respectively. Although they were mostly represented in the annually burned savanna and less represented in the savanna woodland and the forest, their abundance did not vary statistically between habitats, not even between the entire areas investigated. The abundance of wood-feeders did not differ statistically between the two areas investigated. No “dominant” species belonged to this feeding group, although the most abundant wood-feeding species, *Microcerotermes parvus* (Haviland), was found in all habitat types of both areas. In total, this species was represented with 13 occurrences inside the Reserve (25% of the total) and 7 occurrences (35% of the total) in the rural domain. At Lamto, the species was more abundantly collected in the forest than in the two savannas, but it was almost absent in the rural forest. All the grass-feeding species collected belonged to the genus *Trinervitermes.* They were restricted to savannas, and mainly to the annually burned savanna, which provided an elevated quantity of grassy litter. The most encountered and abundant grass-feeder was *T. geminatus*, which attained the level of strong dominance inside the Reserve with 28 occurrences out of 30 (93%) compared to the rural domain, where it was only encountered 12 times.

### Species composition of termite communities

The similarity of the two adjoining areas was assessed by a cluster analysis based on the area-specific termite species composition at transect and habitat levels. This study was completed by the calculation of the complementarity between termite assemblages found in the different habitats. At transect level, the cluster analysis did not show a clear separation between the termite assemblages (Figure not shown). In contrast, at habitat level, three distinctive clusters were formed, each composed of the same habitat type (Figure 3). The assessment of the complementarity showed a high similarity of the termite species compositions of the two areas by sharing 20 species, i.e., 62.5% of their total species richness. Termite species compositions of the two annually burned savannas and the two savanna woodlands were closely overlapping (Table 5). The two annually burned savannas shared 11 species (68.7% of their total species richness), and the two savanna woodlands shared 14 species (70% of their total species richness). Although the Lamto protected forest and rural forest formed similar clusters, complementarity indicated clear dissimilarity between their species compositions. Out of the 25 species found in the Lamto forest, only 12 (48%) were collected in the rural forest. Overall, termite species composition did not change considerably from one area to the other; the two areas (i.e. Lamto Reserve and rural domain) shared 21 species (65.6%) of the 32 species collected.

## Discussion

### Species richness and species diversity of termites

The study showed that the termite assemblage considerably shrinked in the rural forest compared to the Lamto forest, certainly mostly due to various human activities occurring in this habitat (cf. Eggleton et al. 2002). While termite species richness was high in the Lamto forest, it was considerably lower in the rural forest. The pressing need of using forested sites in the rural domain for agriculture, firewood collecting, hunting, and harvest of medicinal plants lead to a reduction of physical complexity of these habitats, causing a decline in the variety and abundance of suitable nesting and feeding sites, as well as changes in microclimate. Many termite species occupy microhabitats such as rotting tree stumps, dead logs, humus around the base of trees and mounds of other species, etc. (Eggleton and Bignell 1997, Jones et al. 2003). Such microhabitats often disappear from an intensively used area. Thus, increasing destruction of rural forests is suspected to reduce the chances of alates finding suitable sites for establishing new colonies and for their growth. Contrary to rural forests, those located inside Lamto Reserve harboured many species and served as refuges for termites sensitive to regular fire occurrences (Dosso et al. 2010). Because of their relative stability, such habitats may provide variable food resources for nourishment and niches for nesting (Grasse 1986). Regardless of the size of the areas (Lamto Reserve and rural domain), the number of termite species did not differ in the annually burned savannas and the savanna woodlands. Among these savanna systems, only the savanna woodland is often used for agriculture, especially when forests become increasingly rare or inaccessible. This habitat type possessed a relatively high number of termite species, certainly due to the settlement of many colonies despite agricultural intensification, a trend that is less evident in forests (Jones et al. 2003). Such a pattern may be explained by the massive appearance of species that are peculiar to this heterogeneous savanna (as *Ancistrotermes guineensis*) and those newly known for the local species pool (as *Ancistrotermes crucifier* and *Pseudacanthotermes* aff. *spiniger*). Otherwise, some species were more tolerant of greater exposure to direct sunlight even if the savanna woodland had been cleared, as with the mound-builder *T. geminatus* (Sands 1965). Most of the grass-feeding species collected in the annually burned savanna of Lamto were found in the equivalent location in the rural domain. As burning events are unpredictable in this latter area, such termites may qualify as highly disturbance-adapted species (Dosso et al. 2010).

### Relative abundance of termites

Significantly more termites were collected inside Lamto Reserve than in the rural domain. This fact could be a consequence of a higher disturbance level in the rural domain, where human pressure on habitats may be unfavorable for natural communities (cf. Brooks et al. 2002). Of the four feeding groups encountered, the fungus-growers were the most abundant group both in the different habitats and in the entire study area. This fact might be favored by the sampling periods (i.e., dry seasons), when biogenic structures (e.g. runways, sheetings) could be used by termites for a longer time than in the rainy seasons. The pairwise comparison of habitat types showed significantly higher abundance of the fungus-growers in the Lamto savanna woodland than in the rural savanna woodland. Such a trend suggested considering other factors than the heterogeneous spatial structure, such as dead wood left on the ground, which enhances recolonization of many termites (Davies et al. 1999). The rural savanna woodland was subjected to human pressure by agricultural activities and the harvesting of firewood, which limited any massive new establishment of termites. While assessing abundances of species, some widely distributed fungus-growing species showed significant differences between the same habitat types in the two different areas. For example, *A. cavithorax* was more abundant in the Lamto savanna woodland and the Lamto forest compared to their equivalent locations in the rural domain. As this “dominant” species has a pronounced ecological plasticity, and thus is a good competitor (Konaté 1998), its decrease suggests that human pressure was the cause for its low abundance in the rural domain. In addition, the species *P. militaris* was significantly more abundant in the annually burned rural savanna and rural forest than in their equivalents located inside the Lamto Reserve. As this species feeds preferentially on living grass, litter, and living wood (Josens 1977), this difference may be attributed to sufficient availability of such plant materials, in spite of recurrent burning practices and live stock eating up the grass in the rural savannas. In all of the considered areas, the abundance of soil-feeding species was surprisingly higher in the annually burned savanna than in the forest. Their “dominant” species (*Adaiphrotermes* sp. and *Aderitotermes* sp.) did not show any significant variation in abundance either between habitats or between the two areas. Although the savannas are poor in organic matter (Abbadie and Nacro 2006), the trend to increased abundances in the annually burned savanna may be explained by the availability of organic matter around and under grass tufts where most species were collected (Dosso et al. 2010). In addition, these grass tufts may offer especially good protection against bush fires representing a major disturbance in this habitat type. Our results agree with that of Tano (1993), who found this feeding group in elevated quantity in the Sudanian savanna of Booro-Borotou (north-western Côte d'Ivoire). As for the savanna woodland, this fact was not observed, as the grass stratum is reduced because of tree invasion. The decrease in abundance of these species in the annually burned rural savanna may be attributed to the high frequency and unpredictability of fires that are too destructive for the vegetation, and thus eliminate the nurture basis of these termites. As for the wood-feeders, their abundance did not vary between habitats or between the two areas altogether. Any soil-feeding species was not collected in such a manner as to attain the “dominance” level. Specifically, species such as *Amitermes* *evuncifer*, Microcerotermes parvus, *Nasutitermes arborum*, *Nasutitermes latifrons*, and *Schedorhinotermes lamanianus* that were relatively well represented in the Lamto Reserve forest were collected in low quantities in the rural forest. This fact may reflect the response of these termites to simplification of the physical structure of the rural forest, which resulted in the reduction of dead wood, the alteration in microclimate, and the loss of nesting sites (Jones et al. 2003). All three grass-feeding species collected belonged to the genus *Trinervitermes.* They seemed to be mainly restricted to the annually burned savanna, where the species *T. geminatus* attained the level of “dominance,” inside the Lamto Reserve. Our observations were congruent with those of Benzie (1986), who found *Trinervitermes* species to be dominant in the same savanna type (Guinean savanna) at Mole National Park, Ghana. With the high grass biomass produced in the annually burned rural savanna, only man's interference (such as frequent fires) may result in a general decrease in *Trinervitermes*' mound densities (Roy-Noël 1978).

### Comparison of species compositions within habitat types

The classification of habitat types based on termite communities resulted in three distinct groups. The first group was composed of the two annually burned savannas possessing species-poor termite fauna (with a total of 16 species). These habitats, which also presented similar species compositions (complementarity = 31%), mainly hosted grass-feeding species (*Trinervitermes* species) and, surprisingly, two dominant soil-feeding species (*Adaiphrotermes* sp. and *Aderitotermes* sp.). The second group comprised the two savanna woodlands, which possessed intermediate species compositions between the annually burned savanna and the forest. With a total of 20 species, they were characterized by their equal species richness. With regard to a heterogeneous spatial structure (i.e., a mixture of trees and grasses), they offer various sources of nourishment and niches for savanna and forest-adapted species (Dosso et al. 2010). Thus, although exploited for agriculture, the rural savanna woodland was highly similar to its equivalent location inside the Reserve (complementarity = 30%). Contrary to the savanna types, the two forests were not similar (complementarity = 59%), although they formed the same cluster in Figure 3. While combining their species compositions, they totaled 26 species but with remarkable unequal species richness. While 25 species were collected in the Lamto forest, only 13 species were found in the rural one. Such a result may be attributed to severe human impact on rural forests, causing a strong decrease in termite diversity and composition and thus in the influence they have on ecosystem processes (Eggleton et al. 2002).

Overall, this study did not reveal major changes in termite assemblages between the Lamto protected area and the adjoining rural domain. The rural savanna systems were closely species-rich and had very similar species compositions as the equivalent habitats located inside the Reserve. This may have resulted from the fact that fire is the common disturbance occurring in the savanna areas, leading to the settlement and the adaptation of the same termites. The markedly affected habitat was the rural forest, which showed a great decrease in termite species richness and a severe modification in the species composition compared to that of the corresponding protected area inside Lamto Reserve. As the objective of this research is to protect Lamto Reserve against negative impacts, the immediate challenge is to assess the impact of agricultural and silvicultural Systems on biodiversity in order to suggest appropriate management practices that protect biodiversity and maintain ecosystem functions.

**Table 1.**
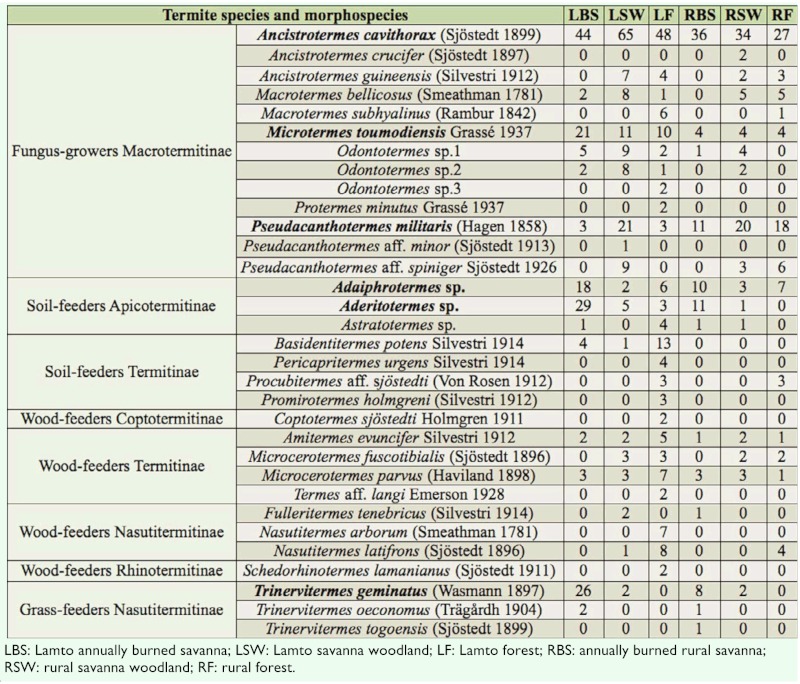
List of termite species collected and number of occurrences in the different habitats. The “dominant” species are written in bold.

**Table 2.**
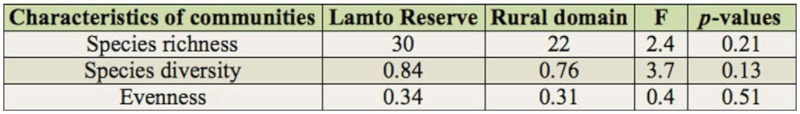
Comparison (using ANOVA 1, ddl = 1) of termite diversity between Lamto Reserve and the rural domain. Significant p-values are written in bold.

**Table 3.**
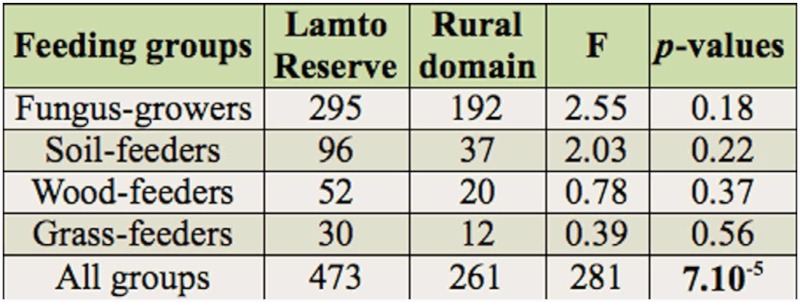
Comparison (using ANOVA 1, ddl = 1) of the relative abundances of termite feeding groups between Lamto Reserve and the rural domain.

**Table 4.**
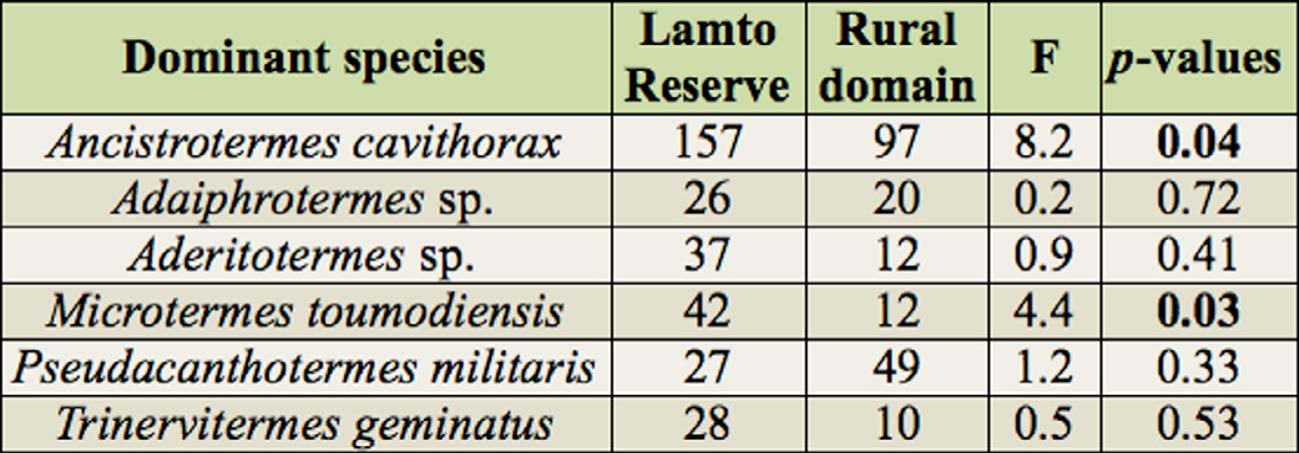
Comparison (using ANOVA 1, ddl = 1) of the relative abundances of dominant species between Lamto Reserve and the rural domain. Significant p-values are written in bold.

**Table 5.**
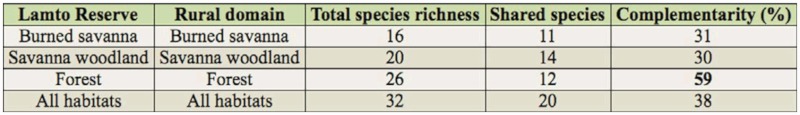
Complementarity of the termite assemblages between habitats belonging to the same type. The value in bold indicates clear dissimilarity between habitat types.

**Figure 2.**
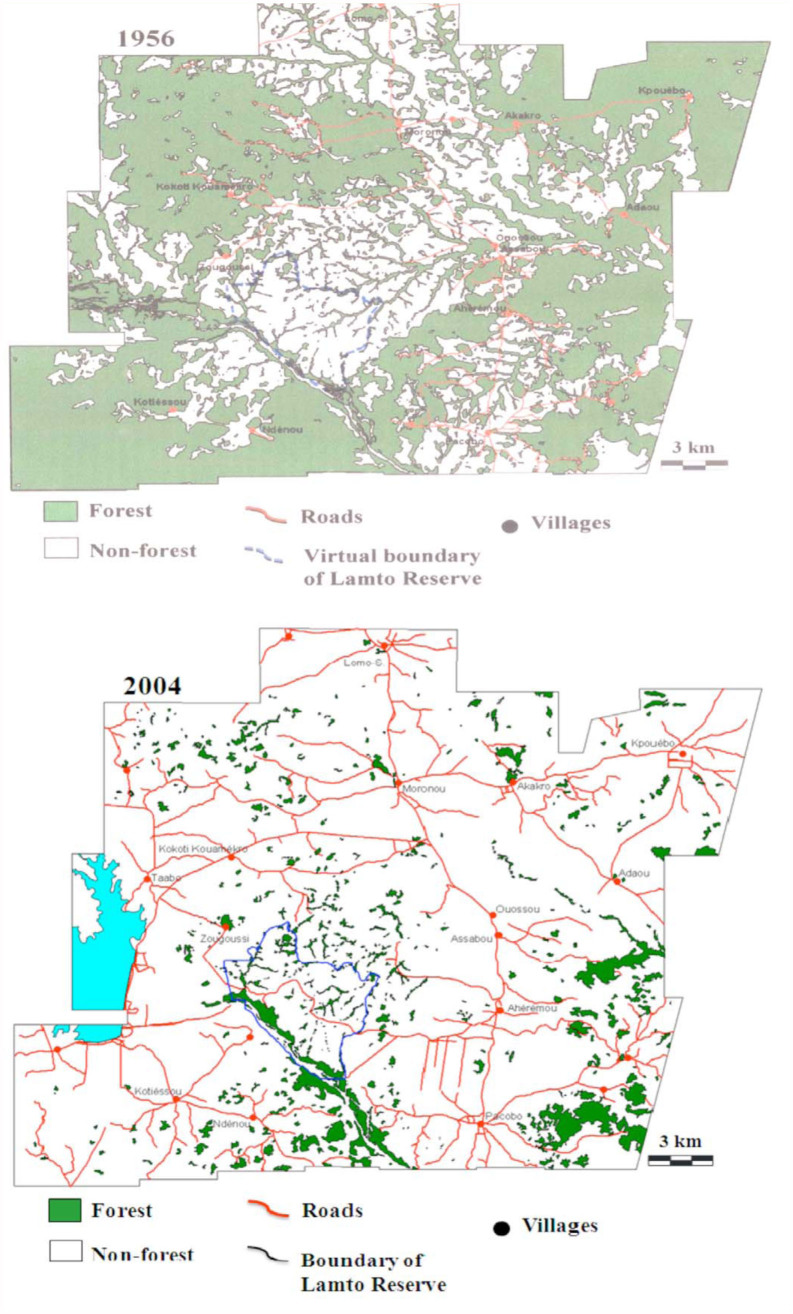
Maps of the Lamto region showing the extent of forest destruction due to human pressure from 1956 to 2004 (1956: Picture digitalized from aerial pictures of the Geographical National Institute of Paris; 2004: Picture digitalized from a panchromatic picture of the SPOT 5, with 5-m spatial resolution) (Goetze and Koulibaly unpublished). High quality figures are available online.

**Figure 3.**
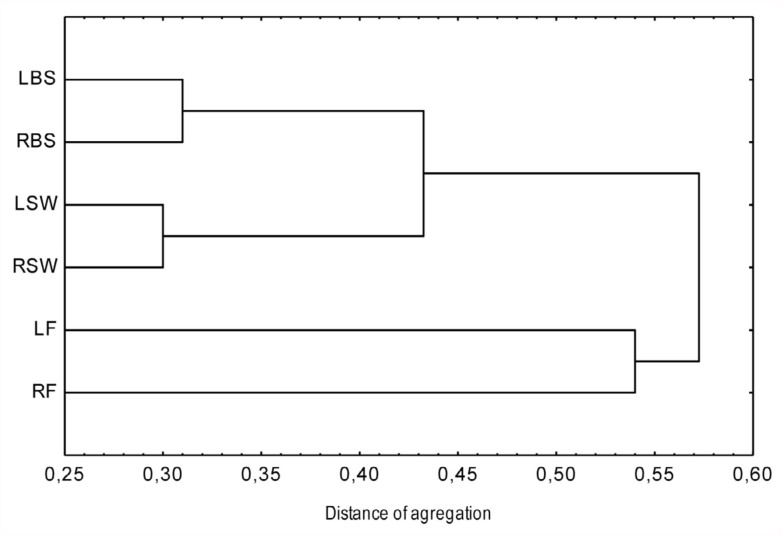
Classification of habitats based on the termite species composition with UPGMA (Unweighted Pair-Group Method using Arithmetic averages) using 1 -Jaccard index as the distance between groups. Abbreviations are defined in Table I. High quality figures are available online.
